# Uniform Fabrication of Hollow Titania Using Anionic Modified Acrylated Polymer Template for Phase Composition Effect as Photocatalyst and Infrared Reflective Coating

**DOI:** 10.3390/nano11112845

**Published:** 2021-10-26

**Authors:** Tae Ho Yun, Changyong Yim

**Affiliations:** 1 Department of Precision Mechanical Engineering, Kyungpook National University (KNU), Sangju 37224, Korea; thyun87@knu.ac.kr; 2 School of Nano & Materials Science and Engineering, Kyungpook National University (KNU), Sangju 37224, Korea; 3 Department of Advanced Science and Technology Convergence, Kyungpook National University (KNU), Sangju 37224, Korea

**Keywords:** titania, hollow structure, photocatalyst, anti contamination, IR reflective coating

## Abstract

Polymer coatings containing thermal blocking and near-infrared (NIR)-reflective pigments have received much attention for their potential applications in energy-saving fields. A drawback of these coatings is sustainability providing similar long-term performance. Surface cleaning is mandatory to remove contaminants that decrease reflectance. In this study, synthesized hollow titania as photocatalyst was used to impart anti-contamination to infrared (IR)-reflective coatings. A TiO_2_ shell was selectively formed on an anionic polystyrene core, modified by methacrylic acid. According to sintering temperature, the enhancement of light absorption ability and photocatalytic activity as methyl orange decomposition was observed with phase composition change. The methylene blue decomposition reaction, reflectance measurement, and measuring thermal profiling of coated steel confirmed the manifestation of hollow particles to dust degradation characteristics and the enhancement of reflection and thermal shielding.

## 1. Introduction

Since Fujishima and Honda reported water splitting from titania electrodes [1], the photocatalytic properties of titania nanomaterials have been widely studied in areas such as water splitting [2,3,4], CO_2_ conversion [5], and organic pollutant degradation [6,7]. Many non-toxic, durable, resistant to reactions with chemical compounds, and cheap photocatalysis (TiO_2_) have been widely studied regarding synthesis, properties, modification, and applications. Nanoscale titania combines the desirable properties of titania with the high surface areas achievable by nanomaterials. Although nano-titania has been used for various applications, its low absorption capability because of wide band gaps and charge recombination in the visible light region limits its use in solar power applications. Many studies have attempted to extend nano-titania absorption to the visible region using different approaches. Doping is a solution and success has been achieved using (noble) metal ions and several atoms [8,9,10,11].

Manipulation of dopant impurity and vacancy bands can improve the efficiency of photocatalysis in terms of mobility and diffusion rate. For example, theoretical study of Cu doping effect on TiO_2_ was reported by density functional theory methods to simulate doped anatase (101) surface plane. Cu doping tends to extend surface-hole life times and increase photo exciton production through effective charge transfer and bandgap narrowing [12]. Cl doping on rutile TiO_2_ nanosheets produces light carriers leading to higher mobility for excitons. The photogenerated electrons and holes extend the lifetime of Ti^3+^, which acts as an in situ cocatalyst [13].

Another approach is the sensitization and semiconductor composite technique. Some dyes with redox properties and visible light sensitivity can be used in solar cells and photocatalytic systems [14,15]. Furthermore, the control effects of the surface properties, sizes, and shapes of titania nanoparticles were reported for increasing photocatalytic activity [16,17]. Mesoporous structure titania increases its light absorption ability because of increased surface area and improved particle light scattering. Moreover, improved light absorption ability enhances surface adsorption of reactants and reinforces photocatalytic activity [18].

Structural dimensionality can also affect the photocatalytic performance and properties of titania materials. Various titania morphologies and structures (spherical particles [19], nanorods [20,21], nanotubes [22], and hollow spheres [23,24,25]) were also studied to enhance the photocatalytic activity of titania nanoparticles. Although there are pros and cons regarding dimensionality, the zero-dimensional structure is better than the one-dimensional structure for the coating system. The one-dimensional structure needs an additional transfer process from ITO (Indium Tin Oxide)to the target substrate for the coating process. Moreover, it needs an arrangement for photocatalytic efficiency. Therefore, the zero-dimensional structure is desired for the coating application to the substrate. The zero-dimensional structure increases the size of the accessible surface area by achieving special structures such as whisker [19] and hollow morphology [24]. Overall, these increases result in better photocatalytic performance because the photocatalytic reaction is based on chemical reactions on the surface of the photocatalyst. Moreover, hollow structural features increase the light-harvesting capabilities of these materials because they enhance light use by allowing as much light as possible to access the interior [23].

Titania hollow spheres can be synthesized using many approaches, including spraying [26], soft and hard template [27,28], sacrificial template [25,29], and hydrothermal approaches [30]. The template method is one of the most widely used ways to construct nanoarchitecture by adequate control of the nucleation and growth. The templates are generally divided into two groups such as hard template and soft template. The soft template approach uses aggregation structural features which are formed by inter and intra- molecular interaction force in the synthesis of nanoparticles. The hard template approach has the advantage of preparation of composite materials with different sizes and architecture under various requirements. In addition, this approach features high stability and reproducibility and can precisely control the dimension and specification of the target material. Metal–organic frameworks (MOFs) have widespread interest in the study of catalysis as hard template candidates due to their porous crystalline property and inherent characteristics toward catalysis. The MOFs are beneficial for highly uniform and tunable pore shape and size—the open pathway used as transport and diffusion [27]. Photocatalytic CO_2_ reduction performance to CH_4_ was achieved by HKUST-1(Cu_3_(BTC)_2_)@TiO_2_ due to efficient exciton transferal from TiO_2_ to the MOF. The MOF provides promoted charge separation in TiO_2_ and active sites for conversion of CO_2_ [28].

Polymer colloidal spherical beads are another commonly used approach in the template method. Titanium precursors are attached to the surface using a layer-by-layer method or sol-gel reaction. However, it is challenging to synthesize titania hollow spheres. Because of the high reactivity of titania precursors, the various methods have several disadvantages, such as the formation of irregular coatings, aggregation of the coated particles, and low efficiency of controlling the coating thickness. Therefore, it is necessary for the formation of dense, smooth titania coatings on the surface of polymer colloid particles to control the hydrolysis and diffusion rates of titania precursors in the reaction system. Controlling the reaction time is challenging because of the high reactivity of titanium precursors.

Typically, the hard template method for forming the core-shell-structured silica or titania particles tends to predominantly use a cationic polymer template. The hard template method involves the (1) preparation of hard templates, (2) functionalization/modification of the template surface to achieve favorable surface properties, (3) coating the templates with designed materials or their precursors using various approaches, possibly with post-treatment to form compact shells, and (4) the selective removal of the templates to obtain hollow structures. Some factors must be considered for the synthesis of well-controlled titania and polymer hybrid composites by in situ polymerizations, such as the (1) compatibility between titania and the polymer, (2) electrostatic attraction, and (3) acid-base interaction. In this study, we synthesized hollow titania spheres of various sizes using anionic modified polymer latex and a SiO_2_/carbon template as core materials using the Stöber method. The photoactivity of the produced particles was evaluated and applied to the paint to confirm the implementation of its capability.

Polymer films containing near-infrared (NIR)-reflective pigments receive attention for their potential applications in energy-saving fields. Organic dust in the air is easily adsorbed and adheres to the surface of these films, gradually reducing their NIR reflectance. Roofs of either white or a light color were cooler than their black counterparts because white has the best solar-reflective performance [31]. However, white or light-colored roofs are readily contaminated by dust in the air, which gradually abates their NIR reflectivity [32,33,34,35]. To overcome the contamination, surface cleaning technology is required to attain sustainability, such as superhydrophobic surfaces that possess self-cleaning properties [36,37]. The removal of contaminants using photocatalyst particles onto the coating could be a candidate. Such a film can reduce the maintenance cost and guarantee equal performance of the IR reflective coating and is dust-proof.

One method for achieving high reflectivity is a multilayer design in which a low refractive index layer (*n_L_*) and a high refractive index layer (*n_H_*) are alternately stacked. Equation (1) gives an overview of the refractive index design of multilayer.

(1) $$E_{refl}=E_{inc}\frac{\left(n_{1}-n_{2}\right)}{\left(n_{1}+n_{2}\right)},\;R={\frac{\left(n_{1}-n_{2}\right)}{\left(n_{1}+n_{2}\right)}}^{2}\;$$

*E* represents the field strength, *R* is the reflectance, refl is the reflection, and inc is the incident. In the above equation, if *n*_1_ > *n*_2_, then the *E_refl_* and *E_inc_* codes coincide, and constructive interference occurs. If *n*_1_ < *n*_2_, then the *E_refl_* and *E_inc_* codes are opposite, so destructive interference occurs. Therefore, to achieve high reflectivity, *n*_1_ > *n*_2_, that is, when the coating layer is applied to the material, the refractive index of the coating layer should be higher than that of the material. A hollow structure is a method of applying the layered design of the refractive index. When hollow particles are introduced, the space inside is air (refractive index = 1), and ceramics, metal oxides, and polymers could be present on the outside. Since these materials have a higher refractive index than air, they can easily reflect light in the long-wavelength region. Furthermore, this method can increase the effectiveness of the insulation. The ceramic, metal oxide, and polymer layers on the outside have a lower thermal conductivity than steel grades used as materials; therefore, they reduce heat transfer because of thermal radiation, and the air inside has the lowest thermal conductivity, which is also great for reducing heat transfer.

In this paper we report that mesoporous hollow structured TiO_2_ nanoparticles were prepared using anionic modified acrylate polymer templates. The effect of acrylate modification on the uniform shell formation with rapid and unstable titania precursors was investigated. The photoactivity of the prepared nanoparticles was investigated using various organic dye materials, especially important properties for anti-contamination. Moreover, the effect of the phase fraction on the photocatalytic ability of the synthesized hollow titania nanoparticles was studied. Furthermore, the effects of the hollow structure were explored in terms of self-cleaning and heat shielding in IR reflective coatings. A suggestion for restraining surface contamination, which is one of the chronic problems of thermal insulation paints, and enhancing the thermal insulation performance was proposed to attain long-term sustainability.

## 2. Materials and Methods

### 2.1. Preparation of the Core Template

The top-down synthesis method was used to synthesize the core-shell-structured and hollow ceramic nanoparticles. Acid-containing core templates were prepared by seed emulsion polymerization using the Stöber method to provide better control over the particle size, produce more monodispersed particles, and minimize the possibility of forming a water-soluble polymer in an aqueous medium. Furthermore, 30 wt% methacrylic acid (99.5%, MAA Samchun Pure Chemical Co., Ltd., Seoul, Korea) was used as an acid functional monomer to make the particles alkali-swellable because of its distribution between the aqueous and monomer/polymer phases favors the latter more than acrylic acid.

For core template 1, the two-stage semi-continuous emulsion polymerization was performed in a 1000 mL, four-necked, round-bottom flask equipped with a paddle stirrer, a thermometer, a nitrogen gas inlet, a reflux condenser, and inlet tubes for the continuous feed of materials. Deionized water (430 g) and 10 wt% sodium dodecylbenzene sulphonate solution (SDBS, Sigma-Aldrich, St. Louis, MO, USA) (6 g) were added to the stirred flask while maintaining a nitrogen atmosphere. Then, a pre-emulsion mixture of styrene monomer (20 g, 99.5%, Samchun Pure Chemical, Seoul, Korea), DIW (10 g), and sodium persulfate (0.48 g, Tokyo Chemical Industry Co., Ltd., Tokyo, Japan) was immediately added to the flask and heated to 80 °C and kept for 30 min. Sequentially, a monomer mixture containing DIW (55.2 g), 10 wt% SDBS solution (2.76 g), and styrene monomer (110.4 g) was fed into the reactor at a rate of 3.74 g∙min^−1^, kept for 1 h, and cooled to room temperature. An additional process was conducted to increase the core latex size. As above, DIW (400 g) and the synthesized polystyrene core aqueous solution (40 g) were poured into a 1000 mL, four-necked, round-bottom flask with a stirrer, a thermometer, a nitrogen gas inlet, a reflux condenser, and inlet tubes for the continuous feed of materials. Sequentially, the solution was heated to 80 °C after injecting sodium persulfate aqueous solution (0.32 g in 8 g DIW). A pre-emulsion mixture of DIW (80 g), SDBS (2.2 g), styrene monomer (100 g), methacrylic acid (25 g), and ethylene glycol dimethacrylate (1 g, Tokyo Chemical Industry Co., Ltd., Tokyo, Japan) was fed into the flask at a rate of 4.07 g/min then, maintained for 1 h to produce an acrylate-modified polystyrene core template, and cooled to room temperature.

For core template 2, a four-neck flask containing DIW (201.6 g), ammonium nonylphenol ethersulfate (0.68 g, Rhodapex CO-436 from Rhodia, Brussels, Belgium), and ammonium persulfate (0.17 g, Sigma-Aldrich, St. Louis, MO, USA) was heated to 80 °C while maintaining a nitrogen atmosphere. A seed monomer mixture consisting of butyl acrylate (BA, 7.4 g), methyl methacrylate (MMA, 3.25 g), and methacrylic acid (MAA, 0.14 g) was rapidly charged into the flask and maintained for 1 h. The sodium persulfate aqueous solution (0.76 g in DIW 42.3 g) and ammonium nonylphenol ethersulfate solution (1.21 g in DIW 120.7 g) were then added to the flask. A monomer mixture of butyl acrylate (5.49 g, Samchun Pure Chemical, Seoul, Korea), methyl methacrylate (MMA, 78.8 g, Samchun Pure Chemical, Seoul, Korea), methacrylic acid (38.2 g), and hexanediol diacrylate (HDDA, 1.58 g, Tokyo Chemical Industry Co., Ltd., Tokyo, Japan) was fed into the reactor at a rate of 2.08 g/min, and the reaction temperature was lowered to room temperature after a 1 h reaction.

For core template 3, aqueous SDBS solution (0.2 g in 140 g DIW) and sodium persulfate (0.15 g in DIW) were added to the flask and heated to 80 °C. Styrene (3.2 g), SDBS (0.008 g), and water (1.6 g) were added to the flask for 1 min and maintained for 25 min at 80 °C. Styrene pre-emulsion (styrene (36.8 g), SDBS (0.092 g), and DIW (18.4 g)) was fed for 1 h, maintained for another 1 h, then cooled down to room temperature. In sequence, aqueous polystyrene latex solution (25 g in 198 g DIW) was added to the reactor, followed by 0.16 g SPS in 2 g of DIW. Monomer mixture pre-emulsion was fed for 1 h and maintained for another 1 h before cooling. The monomer mixture comprises MMA (35.9 g), MAA (14.9 g), HDDA (2.5 g), CO-436 (0.21 g), and DIW (40 g). Silica deposition was conducted using a modified Stöber method that involved the hydrolysis and condensation of tetraethyl orthosilicate (TEOS, Tokyo Chemical Industry Co., Ltd., Tokyo, Japan), a commonly used sol-gel precursor to amorphous silica. A desired amount of TEOS was added in batch or semi-batch mode to the core solution containing ammonia. In a typical synthesis, 2.5 g (30 wt%) of synthesized acrylate-modified polystyrene core solution was diluted with ethanol and deionized water. Under magnetic stirring, an ammonia solution was added to the batch reactor at room temperature. The TEOS and ethanol mixture solution was fed into the reactor at a rate of 0.57 g/min (feed time is 45 min). The reaction was conducted at room temperature for 8 h under stirring.

### 2.2. Preparation of Titania Hollow Spheres

Three different templates (two carbon templates (cores 1 and 2) and one SiO_2_/carbon template (core 3)) were dissolved in ethanol (99.5%). *N*,*N*-Dimethylethanolamine (DMEA, 99%, Acros Organics, Waltham, MA, USA) or ammonia solution (28–30%, Samchun Pure Chemical Co., Ltd., Seoul, Korea) were added to the template-ethanol solutions under vigorous stirring for 30 min. Titanium tetrabutoxide (TBOT, 99%, Sigma-Aldrich, St. Louis, MO, USA) was quickly poured into the template-ethanol solutions. The template-ethanol solutions containing TBOT were stirred for 6 h. The core-shell-structured template particles coated with TBOT were separated by centrifuging, and the residue was discarded. The separated template particles were placed in a 60 °C convection drying oven for 1 day. Next, the core-shell-structured TiO_2_ composite particles were gathered and calcined at varying temperatures from 400 to 800 °C for 2 h with an increase of 10 °C/min in air to obtain the hollow titania spheres. Table 1 lists the detailed synthesis formulation.

### 2.3. Coating Application

Galvanized steel (POSCO) was degreased with surface-cleaning chemicals and deionized water. Synthesized hollow titania pigmented coating was applied to the steel for evaluating photocatalytic performance and the physical properties of titania. Solvent-borne thermal insulation paint (non-volatile ~40) of fluorine-based resin (Noroo paint & coatings, Anyang, South Korea) was applied on the galvanized steel using a bar applicator and cured at peak metal temperature (PMT) of 224 °C in a convection oven. The dry film thickness was measured using a portable thickness measurement system (Quanix 8500, Automation Dr. Nix, Köln, Germany), and the thickness was 25 μm. Hollow titania pigmented coating was prepared using the same paint as above by mixing pre-dispersed hollow titania solution in ethylene glycol dibutyl ether (non-volatile ~40).

For preparing the clear coating, the main binder was polyester resin (Skybon ES-960 from SK Chemical, Seongnam, Korea). Trixene-blocked isocyanate (BI7982 from Baxenden Chemicals Ltd., Lancashire, UK) was used as a curing agent. p-Toluenesulfonic acid (Nacure 2530 from King Industries Specialty Chemicals, Norwalk, CT, USA) and dibutyltin dilaurate (Sigma-Aldrich, St. Louis, MO, USA) were used as catalysts. Kokosol 150 (SK Chemical, Seongnam, Korea) was used as a solvent. A bar applicator was used to apply the coating to the substrate and the film was cured at PMT of 220 °C in a convective curing oven. Detailed formulation was summarized in Table 2. The dry film thickness was measured using a portable thickness measurement system (from Quanix 8500, Automation Dr. Nix, Köln, Germany), and the thickness was 26 μm.

### 2.4. Characterization

The surface morphology of the synthesized titania (core-shell and hollow) was observed using scanning electron microscopy (SEM, Hitachi SU-6600, Tokyo, Japan) with an accelerating voltage of 20 kV. Before SEM observations, the samples were coated as pretreatment with 10-nm Pt/Pd. Transmission Electron Microscope (TEM, JEOL-2200FS, Tokyo, Japan) measurements were conducted using an image Cs-corrector with an accelerating voltage of 200 keV. The phase analysis and composi-tion determination of hollow titania was conducted using an X-ray diffractometer (XRD, D8 advance, Bruker AXS, Billerica, MA, USA) with CuKa radiation (0.154 nm). The Brunauer–Emmett–Teller (BET, BELSorp-Max, MicrotracBEL Corp, Osaka, Japan) surface area and Barret–Joyner–Halenda (BJH) pore size distribution measurements were conducted using N2 as the adsorptive gas. Various light responses of synthesized particles and pigmented steel sheets, such as light absorption performance in the UV region and light reflectance in NIR/MIR wavelengths, were measured using a UV–vis-NIR spectrophotometer (UV-3600 Plus, Shimadzu, Kyoto, Japan) with integrating sphere attachment (ISR-2600Plus, Shimadzu, Kyoto, Japan). Diffuse reflectance was measured by placing the measurement sample next to the reflectance measurement window on the side of the integrating sphere with a barium sulfate white plate as a standard to obtain the relative diffuse reflectance values.

The photocatalytic activity of the prepared titania particles was evaluated by degrading 20 mg/L methyl orange solution in a reservoir. Titania colloidal aqueous solution (0.5 g/L) was placed in the reservoir (50 mL). A photocatalytic degradation reaction under stirring was the conducted using UV irradiation after 30 min in the dark. The UV irradiation was provided by a 200 W mercury-xenon lamp (Execure 4000-d, Hoya candeo optronics Co., Saitama, Japan). Sampling was performed at regular intervals during the reaction. The residue concentration of methyl orange was calculated by measuring its absorbance at 460 nm using UV–vis-spectroscopy (UV-2501PC, Shimadzu, Kyoto, Japan) [38]. The decomposition rate was calculated using the absorbance ratio to the initial absorbance value. Moreover, the photocatalytic activity observation of a hollow titania coated steel sheet was conducted in methylene blue solution (20 mg/L) under UV irradiation, as above. A coated steel sheet (50 mm × 50 mm) was prepared for the specimen. Methylene blue solution sufficiently covering the specimen surface was dropped on the steel sheet. Visual observation analysis was conducted at regular intervals under UV irradiation.

Particle migration (or colloidal stability) analysis in the solution was conducted using the Turbiscan lab (Lean on tech) for 10 min. The intensity of both transmitted and backscattered light covered the entire solution height when an emitted light, scattered by particles, passed through the sample solution. These intensities allowed the direct monitoring of local physical heterogeneities with a vertical resolution. Thus, the nascent destabilization phenomenon (sedimentation or creaming layers, aggregates, agglomerates, or coalescence) could be detected and monitored periodically at different intervals.

**Figure 1:**
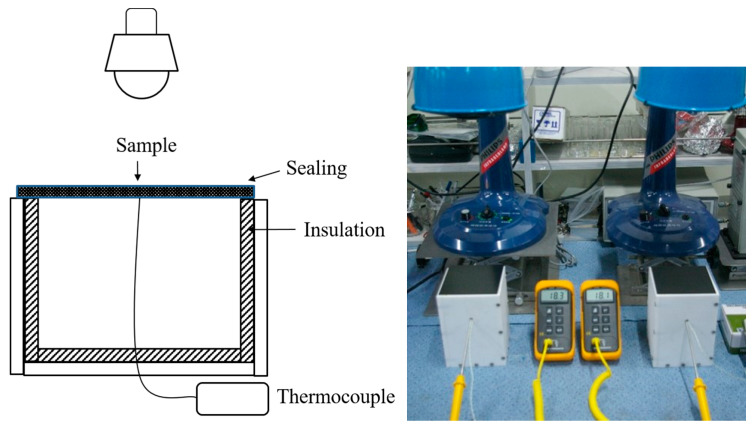
ASTM D4803 modified device for evaluating the IR reflectance of the coated steel sheet.

The thermal blocking performance of coating was evaluated using a modified ASTM D 4803 (standard test method for predicting heat buildup in polyvinyl chloride (PVC) building products) method under 250 W IR irradiation (IF-100, Philips, Amsterdam, Netherlands). A sealed Teflon box (160 mm × 160 mm × 250 mm, top-opened) with thermocouples was prepared instead of an open PVC box system of ASTM D 4803 (Figure 1). A 150 mm × 150 mm coated steel sheet was placed on top of the box and sealed with Teflon. The temperature on the backside of the two coated steel sheet specimens was measured simultaneously to minimize environmental effects.

## 3. Results

### 3.1. Synthesis of Hollow Titania

**Figure 2:**
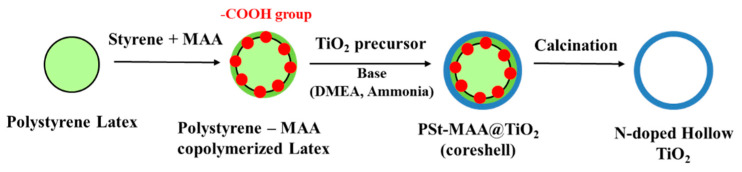
Schematic diagram of the synthesis of core@shell titania and N-doped hollow titania nanoparticles.

The polymer capsules were synthesized by two-stage emulsion polymerization, namely, preparing the seed latex particle and forming the shell layer on the seed latex with BA, MMA, MAA, and HDDA. MAA was used in a seeded semi-continuous emulsion polymerization for proper control over mono-dispersity, particle size, and reducing an inhomogeneity polymer. Moreover, MAA, an acid functional monomer, has better stability in the monomer/polymer phase than acrylic acid. However, acrylic acid tends to remain in the aqueous phase where it can form a water-soluble inhomogeneous polymer. The acid-containing shell provides negative charges in base pH conditions because of the carboxylic group and dispersion stability of its particles. Uniform core-shell-structured titania formed on the PS-MAA and PMMA-MAA core templates (Figure 2).

**Figure 3:**
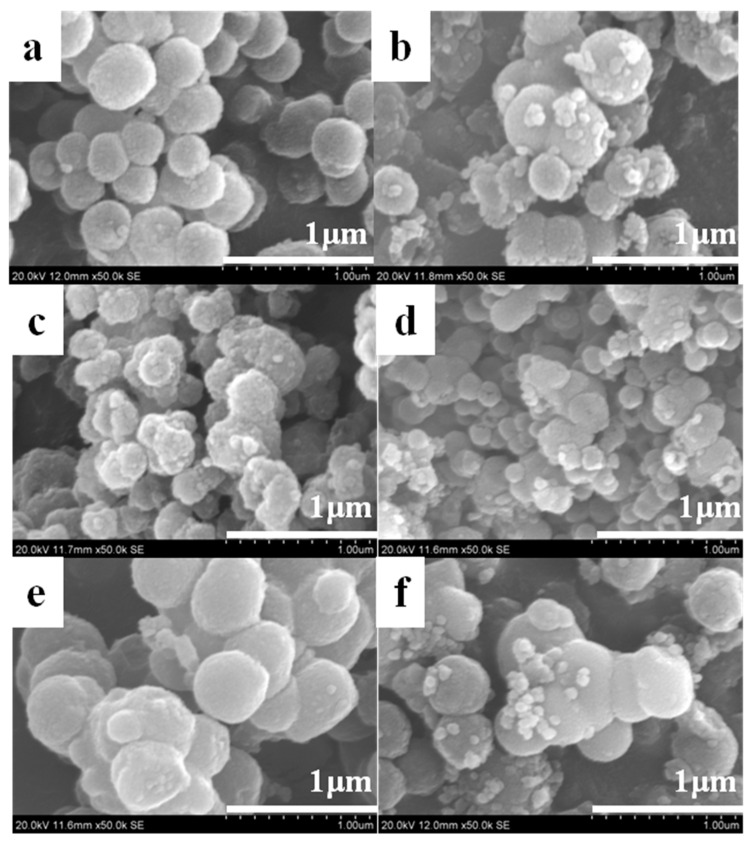
SEM micrographs of synthesized titania: (**a**) CS1 (before calcination); (**b**) CS1 (after calcination); (**c**) CS2 (before calcination); (**d**) CS2 (after calcination); (**e**) CS3 (before calcination); (**f**) CS3 (after calcination).

Figure 3 shows the SEM images of the synthesized titania particles. Figure 3a,c shows the core-shell-structured titania and uniform titania spheres, respectively. After calcination (Figure 3b,d,f), most particles retained their original structure. The titania layer seemed sturdy and well-suited to protecting the shell structure from collapse during the high-temperature heat treatment. Titania layers (core-shell-structured and hollow) formed on the surface of the core template, even though the core type and size differed. An organic carbon template was used to synthesize CS1 and CS2, whereas an inorganic silica/carbon template was used to produce CS3. The selective growth of titania precursors occurred, regardless of whether the template was organic or inorganic.

**Figure 4:**
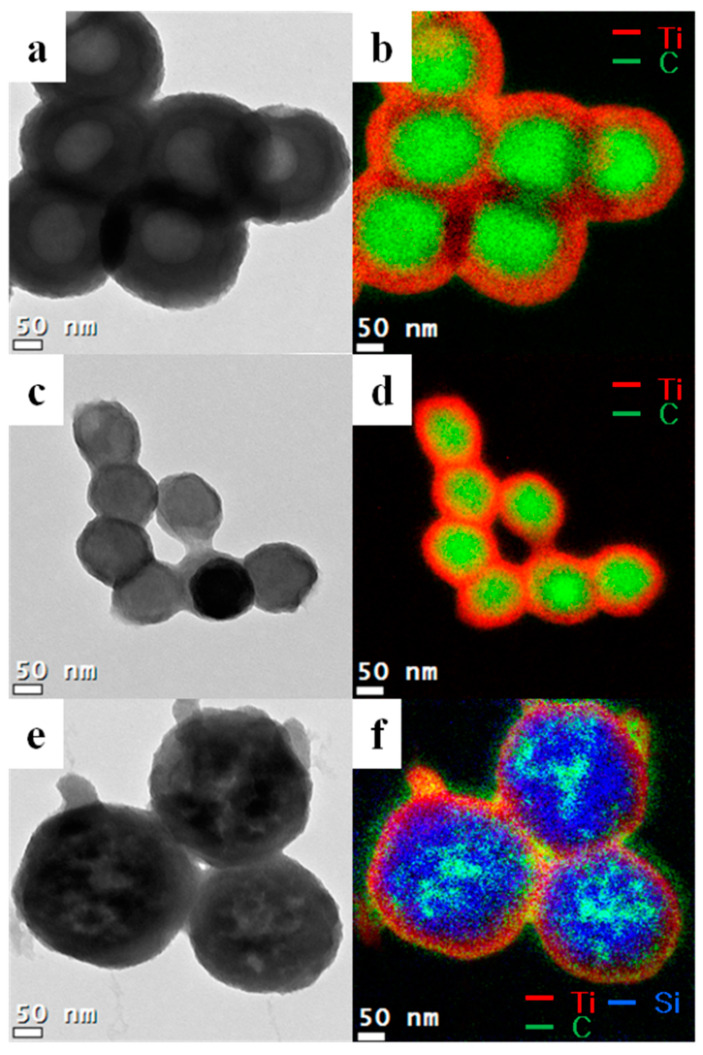
TEM images of core-shell-structured titania spheres (**a**) CS1; (**c**) CS2 and (**e**) CS3. Electron energy loss spectroscopy (EELS) mapping of synthesized titania (**b**) CS1; (**d**) CS2 and (**f**) CS3. The red lines show the Ti atoms, green lines show the carbon atoms, and blue lines show the silicon atoms.

Figure 4 shows the TEM images of core-shell-structured titania. Varying sizes of core-shell-structured titania were synthesized using different core templates, and the average thickness of the titania layer on the core template was ~50 nm. The template sizes were 200 nm for core 1 (CS1), 100 nm for core 2 (CS2), and 250 nm for core 3 (CS3). Core 3 is comprised of silica and carbon materials. However, the titania and SiO_2_ layers were too thick to detect the carbon atoms’ energy loss. A small portion of the carbon region could be observed instead, and the CS3 titania appeared porous. As mentioned above, titania precursors or fine particles were selectively attached to the core template.

**Figure 5:**
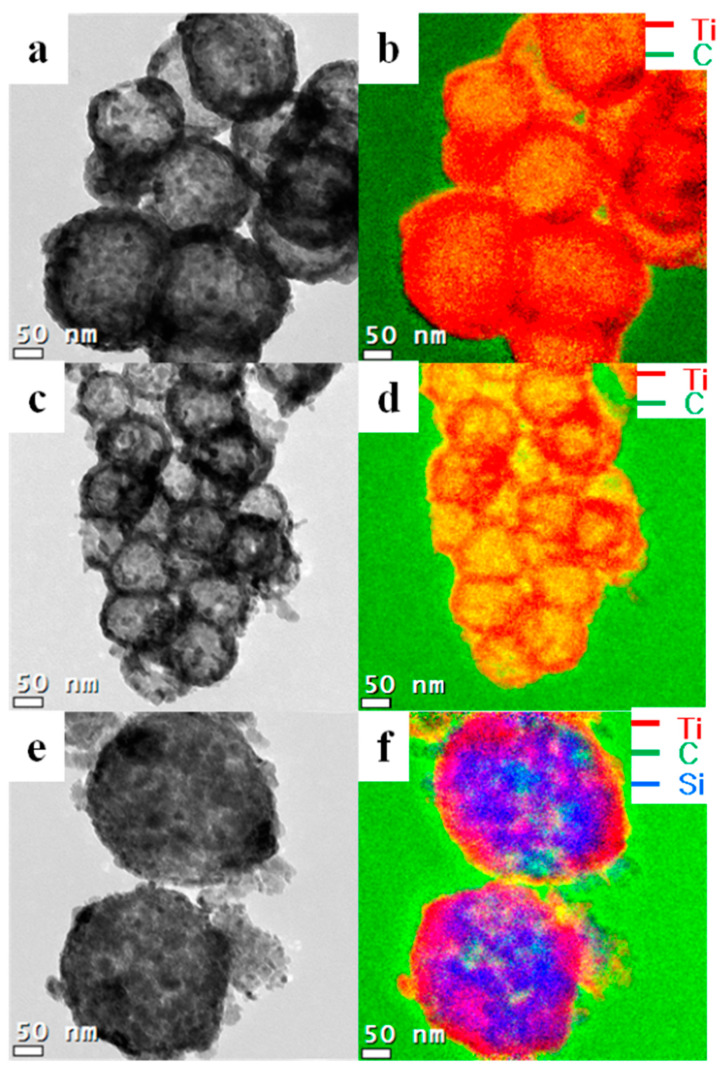
TEM images of core-shell-structured titania spheres (**a**) CS1, (**c**) CS2, and (**e**) CS3. Electron energy loss spectroscopy (EELS) mapping of synthesized titania (**b**) CS1, (**d**) CS2, and (**f**) CS3. The red lines show the Ti atoms, green lines show the carbon atoms, and blue lines show the silicon atoms.

Figure 5 shows the TEM images of hollow titania spheres, indicating that the carbon materials were selectively decomposed by high-temperature calcination, forming mesoporous hollow titania particles. The transmission and differing intensities revealed the mesoporous hollow structure shown by the green carbon light between the grid and titania. The green light of the copper grid was intense because of the absence of carbon in titania. The void sizes of the hollow titania spheres were ~140, 80, and 230 nm for CS1, CS2, and CS3, respectively. The void sizes decreased after calcination because of volume shrinkage during calcination. The titania shell thickness was also reduced to 30–40 nm for the same reason. Overall, these results indicate the hybridization of the two materials containing hollow structures. As mentioned above, one of the principles should be considered for the synthesis of well-controlled core-shell titania, namely, the compatibility between silica and polymer, electrostatic attraction, and acid-base interaction [39]. Using the negative surface charge of silica particles, initiators providing positive charges to the polymer surface increase the incorporation of silica particles [40,41].

However, uniform coatings of titania on anionic PS spheres have also been reported, by using ammonia catalyzed hydrolysis of titanium tetrabutoxide in mixed solvents [42]. The core-shell titania was prepared on anionic polystyrene particles that were synthesized by emulsifier-free emulsion polymerization using potassium persulfate (KPS) as the anionic initiator. The role of ammonia as a catalyst was emphasized. The mechanism indicated that positively charged ammonium ions are formed on the surface of the cationic polymer template, and uniform core-shell particles can be formed because of the negatively charged titania precursor and electrostatic attraction. In this study, PS and PMMA modified with methacrylic acid were used as a template. They are dissociated under neutral/basic pH conditions and exist in carboxylate salt form because the pKa of methacylic acid is 4.8. The prepared polymer template is anionic. Similarly, the amino-based catalysts (ammonia and DMEA), which control the hydrolysis and condensation rate in the sol-gel reaction, are present in the form of a salt with a positive charge at high pH. A uniform and stable core-shell particle is formed because of an electrostatic attraction between the negatively charged titania precursor and the positively charged surface of the polymer.

Furthermore, the effect of the carboxylate salt itself can be considered. Some studies used a sulfonated polymer template to prepare hollow titania spheres [43,44]. The sulfonic acid pendant on the polystyrene chains and the sulfone groups introduced on both inter- and intra-polystyrene chains caused the hydrophilic and cross-linking properties of the hydrogel. Because of the static electricity of the sulfonic acid group and the template, the titanium precursor can be favorably adsorbed and hydrolyzed in the shell, forming titania–PANI/PS composite capsules. Although few reports on the effect of methacylic acid use or the treatment of polymer for the synthesis of core-shell silica or titania exist, it should play a similar role as the sulfonic acid treatment-modifying surface property of polystyrene from hydrophobic to hydrophilic and be a driving force of titania precursor to the template.

**Figure 6:**
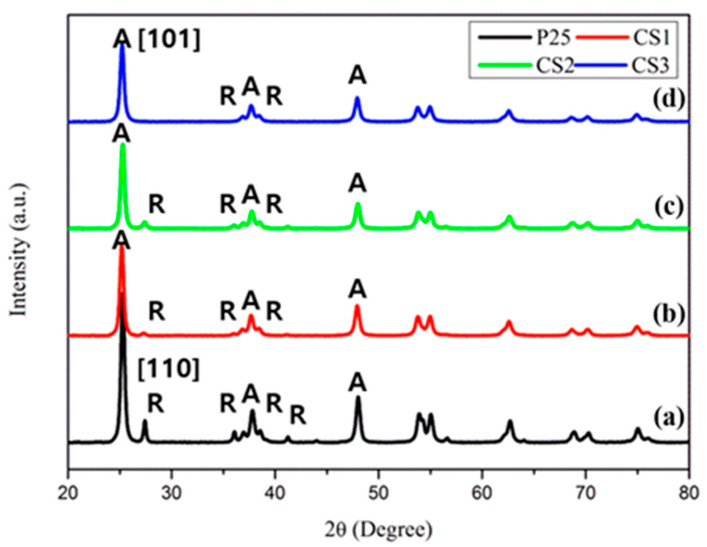
X-ray diffraction (XRD) patterns of P25 and hollow structured titania spheres: (**a**) P25; (**b**) CS1; (**c**) CS2 and (**d**) CS3. Calcination temperature: 700 °C.

Figure 6 shows the XRD patterns for P25 and hollow titania nanoparticles. The XRD patterns indicate the crystallinity of the hollow titania. Most of the hollow titania consisted of a primary anatase phase and a small rutile phase. The average crystallite size of each powder was calculated as ~20 nm using the Debye–Scherrer equation (Table 3). Synthesized hollow titania particles were composed of more than 90% of the anatase phase. The anatase portion decreased as the particle sizes for CS1 and CS2 decreased. The crystallized rate increased during heat treatment for small size particles. Moreover, the A/R phase composition changed according to the core template. CS3 shows the highest anatase composition (~99%). The phase composition from anatase to rutile at 700 °C was delayed with the presence of the silica core. The photocatalytic activity of titania differs from the anatase and rutile phases. Therefore, the phase composition of synthesized particles affects photocatalytic activity and is discussed later.

**Figure 7:**
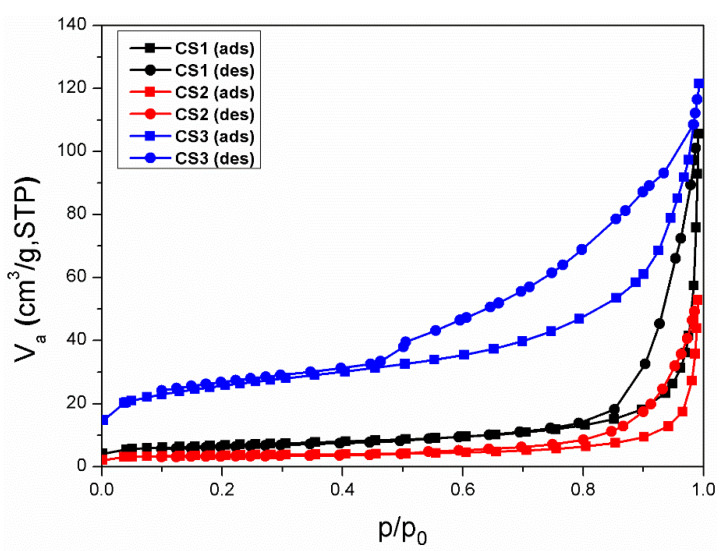
N_2_ adsorption-desorption isotherms of synthesized hollow titania nanoparticles.

Figure 7 shows the N_2_ adsorption-desorption isotherms of the hollow particle. Both samples exhibited type-IV isotherms. The initially abrupt increase at P/P_0_ ~0.05 further indicates the completion of monolayer coverage and initiation of multilayer adsorption. Table 3 summarizes their surface areas. Titania nanoparticles (anatase, diameter: 150 nm) were used as a reference particle. The surface area of the synthesized particle is higher than the reference particle. The hollow structure provides an additional reaction site in the inner space and increases the surface area. For CS3, the surface area is high comparable to the others because of the core template. The silica core template is highly porous, thus the hollow SiO_2_@Titania particle is also highly porous. However, it does not mean an improvement of the titania surface area. Table 4 calculates the phase fraction of the synthesized particles while the heating calcination treatment temperature is from 400 to 800 °C. The table shows that the phase fraction of anatase and rutile changes depending on the temperature of heat treatment. As before, it shows that the smaller the size of the template, the faster the conversion from anatase to rutile phase is. The results of CS1 and CS2 show that the phase transition of anatase/rutile occurs rapidly at 700–800 °C. To change the phase fraction using this, heat treatment must be performed in the corresponding temperature section. Interestingly, when silica is present in the template, such as CS3, the phase transition to rutile transformed slowly; therefore, silica slows the crystallization rate of titania and the phase transition.

### 3.2. Light Response Ability and Photocatalytic Activity of Hollow Titania

**Figure 8:**
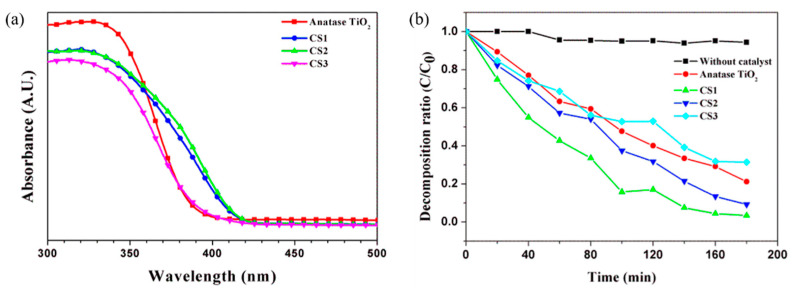
(**a**) UV–vis absorption spectrum and (**b**) photocatalytic methyl orange degradation of synthesized hollow particles (calcination temperature: 700 °C) under UV light irradiation time.

UV–vis absorption measurement and organic pollutant decomposition reaction (methyl orange decomposition) were conducted to characterize the photocatalytic properties of the synthesized particles. Previously, core-shell-structured titania particles were prepared using a sol-gel reaction on various acrylic acid-modified polymer templates. The multiphase titania with various phase compositions could be produced during heat treatment to obtain the hollow structure with different polymer templates. Figure 8 shows the absorption and methyl orange decomposition ability of hollow titania (CS1, CS2, and CS3) prepared by heat treatment at 700 °C. Commercial anatase titania particles show high absorbance in the UV region (λ < 400 nm), which is a characteristic of anatase titania particles with a bandgap of 3.0–3.2 eV, showing light absorption up to a wavelength region of 375 nm. The hollow titania nanoparticles can absorb light in a wider wavelength range than the absorption region of conventional anatase titania. Both CS1 and CS2 show the ability to absorb light to 410 nm because of a multiphase effect (rutile proportion of 15–25%). However, for CS3 (SiO_2_@Titania) particles, the absorbance spectrum is like that of commercial anatase titania because of the formation of anatase phase dominant particles, even though a heat treatment process at 700 °C, and the proportion of rutile is low. A hollow structure can increase the efficiency of the photocatalyst because of an increase in both the available wavelength range and surface area.

**Figure 9:**
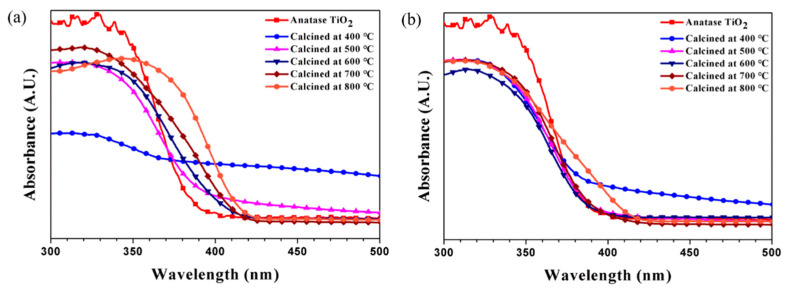
Absorption spectrum of titania hollow spheres with different calcination temperatures under UV light: (**a**) CS1 and (**b**) CS3.

The MO decomposition experiment confirms the above (Figure 9b). The organic decomposition rate is excellent for the CS1 particles. Because of the high surface area, high photoactive efficiency can be achieved by increasing the reaction site, even though the light absorption capability of CS1 and CS2 is similar. The photocatalytic degradation of an organic compound such as dye can generally be explained on the basis of the Langmuir–Hinshelwood process considering the production of electrons and holes produced by the photoexcitation of the catalyst. The absorption of photons induces to the excitation of electrons from the valence band to the conduction band generating electron–hole pairs. The electron in the conduction band is captured by oxygen molecules dissolved in an aqueous media and the hole in the valence band can be captured by OH^−^ or H_2_O species adsorbed on the surface of the catalyst to produce the reactive oxygen species (ROS), such as singlet oxygen (^1^O_2_), hydroxyl radicals (OH•), and super oxide (O_2_^−^). The ROS produced in the above manner can then react with the dye to form other species and is thus responsible for the discoloration of the dye. It may be noted that all these reactions in photocatalysis are possible due to the presence of both dissolved oxygen and water molecules. The photocatalytic degradation of an MO is believed to take place according to the following mechanisms:

(2) $${\mathrm{TiO}}_{2}+h\nu \;\to h_{vb}^{+}+e_{cb}^{-}$$

(3) $$O_{2}+e_{cb}^{-}\;\to \;•O_{2}^{-}$$

(4) $$H_{2}O+h_{vb}^{+}\to \;•\mathrm{OH}+H^{+}$$

(5) $${\mathrm{OH}}^{-}+h_{vb}^{+}\to \;•\mathrm{OH}$$

(6) MO (dye)+•OH→ degradation products

MO seems to decompose stepwise, firstly to N, N-dimethyl-p-phenylenediamine (C_8_H_12_N_2_) and sulfanilic acid (C_6_H_6_NSO_3_) and then N-dimethylamino phenol (C_8_H_11_NO), N-methyl benzene amine (C_8_H_11_N), benzenesulfonic acid (C_6_H_6_O_3_S), and aniline.

Figure 9 shows the absorption spectrum of titania hollow spheres at different calcination temperatures. In Figure 9b, the absorption of CS3 particles does not change with the calcination treatment temperature because of the slight change in the proportion of rutile phase in the crystalline fraction. For CS1, the absorption spectrum varies with the A/R crystal phase fraction via the heat treatment temperature. The particles heat-treated at 400 °C are amorphous and show a low but broad absorbance. Although the wavelength range for the absorbing light is wide, it is unlikely to have any benefit for photoactivity because crystallization has not been achieved. In the case of particles heat-treated at 500–800 °C, the absorption increases as the content of rutile increases. Particles (A/R ~2/98) heat-treated at 800 °C have absorbing light ability in the broadest wavelength range among the calcinated samples.

**Figure 10:**
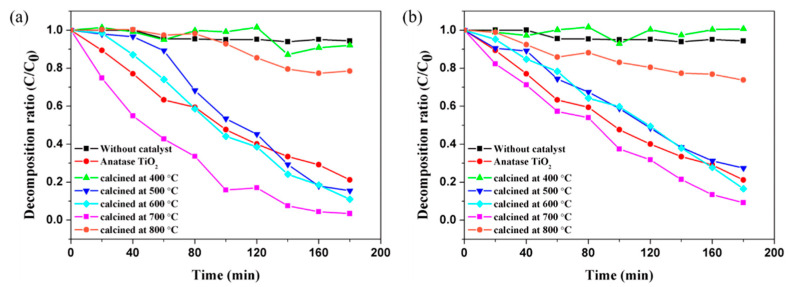
MO decomposition rate of different calcination temperatures (**a**) CS1 and (**b**) CS2.

Figure 10 shows the MO decomposition ratio via different calcination temperatures. Both CS1 and CS2 have different photoactivity during heat treatment temperature conditions. Because the A/R fraction affects the MO decomposition rate, an optimized calcination process is required to maximize the performance. In this study, an excellent photocatalytic effect was confirmed when the rutile content was 15–25% through a heat treatment process of 700 °C. However, it is challenging to assure that only high absorbance enhances the photocatalytic properties because of the 800 °C calcinated particles. Because the rutile content is high, the absorbance spectrum has broad light absorbance in the wide range. The ability to decompose organic pollutants is poor from the MO decomposition rate. The surface area can also affect photocatalytic capability. The increase in the surface area because of the hollow structure indicates an increase in more photoactive reaction regions. Therefore, the complex effect of the hollow structure and phase fraction between particles affects the enhancement of photocatalytic ability.

**Figure 11:**
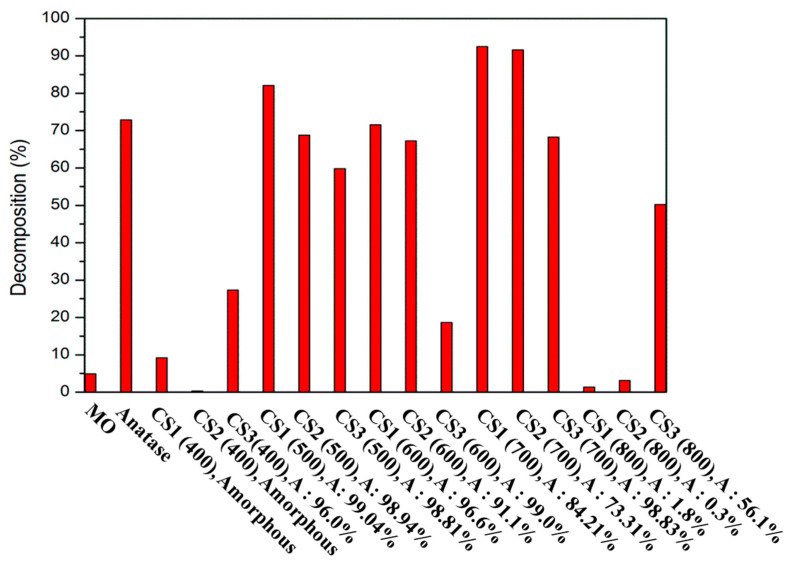
MO decomposition comparison of different calcination temperatures and phase composition of synthesized hollow particles.

Figure 11 shows the decomposition efficiency of organics obtained by conducting MO decomposition experiments on hollow titania particles via heat treatment temperature. The decomposition efficiency was calculated by the concentration of the remaining MO through UV irradiation for 3 h. In the case of MO, it showed a decomposition capacity of ~5%, and in the case of similar efficiency, it was observed at a high ratio (>90%) of amorphous and non-crystallized amorphous and rutile. Furthermore, in the case of particles heat-treated at 800 °C, CS1 and CS2 have low anatase fractions, and in the case of CS3, ~50% anatase fractions. In this case, CS3 shows high photoactivity, indicating that the particles show photoactivity only when a fraction of anatase is present. Samples heat-treated from 500 to 700 °C show high fractions of anatase and different fractions of rutile from 0 to 30%. By adjusting the high anatase fraction and rutile ratio, the photoactivity of the catalyst is changed, which can be explained by the synergy effect of anatase and rutile. The rutile phase has Ti^3+^ ions, functioning as electron donors, which helps to separate the electron holes, and prevents the recombination of electron holes, effectively helping electrons migrate to the titania surface. The mixture of anatase/rutile shows high photoactivity; therefore, it can be explained that P25 shows high photoactivity. The case showing the highest decomposition efficiency is the case of heat treatment at 700 °C. Here, CS1 shows the highest photolysis efficiency, and CS2 and CS3 show higher decomposition efficiency than particles heat-treated under different conditions. Through this experiment, it was found that the condition exhibiting high photoactive efficiency was achieved by controlling the phase fraction.

### 3.3. Evaluation of Light Activity of Synthesized Hollow Particle Coated Film

The buoyant capability of synthesized titania particles in the coating was characterized by dynamic light scattering analysis of the solution. Multiple scattering of particles in dispersion was formed by a light source at 880 nm in a Turbiscan lab. According to the following formulas, the backscattered light (BS) represented dispersion stability of a concentrated sample and varied with concentration changes.

(7) $$\mathrm{BS}=\frac{1}{\sqrt{\lambda^{*}}}\;$$

(8) $$\lambda^{*}\left(\varphi ,d\right)=\frac{2d}{3\varphi \left(1-g\right)Q_{s}}\;$$

where λ* is the mean free path of photons in the disperse system, *φ* is the volume fraction of particles, *d* is the mean diameter of particles, and *g* and *Qs* are the optical parameters given by the Mie theory. The transmission or backscattering measured by each detector depends on the mean free path (λ*) of photons, whereas photons cause scattering with multiple particles within the dispersion. The mean free path of these photons is determined by the size (*d*) and concentration (*φ*) of the dispersed particles. Therefore, the backscattering profiles as a function of the sample height could reflect the microscopic characteristics of growth or migration of particles at a given time.

**Figure 12:**
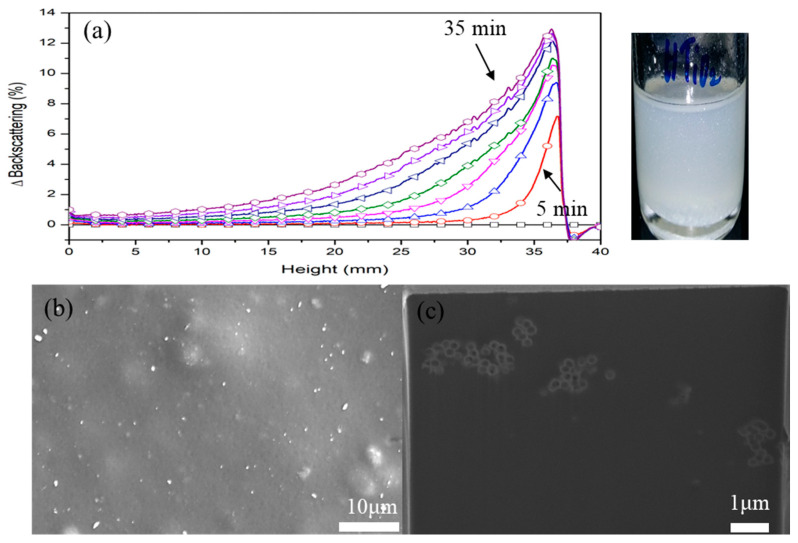
(**a**) BS profiles of titania aqueous solution (0.05 g/L) within 35 min; (**b**) top and (**c**) cross-sectional images of coating containing hollow titania.

Figure 12a shows BS profiles of titania aqueous solution (0.05 g/L) within 35 min at room temperature. Dispersion stability changes of liquid dispersion decrease with time under a fixed temperature condition and are divided into particle migration (creaming, sedimentation) and particle size variation (flocculation, coalescence) phenomena because of the aggregation of dispersed merchant particles. When the particle migration phenomenon, such as creaming or sedimentation, decreases the dispersion stability, the concentration of the entire sample should not change from the initial uniform dispersion state, whereas a concentration gradient can be created between the upper and lower parts of the sample. Scattered transmission or backscattering then causes a local variation of the sample. Moreover, particle size variation phenomena, such as flocculation or coalescence, reduce dispersion stability. Here, because the particle size of the entire sample changes, whole scattering transmission or backscattering also changes to the height of the entire sample.

Particle migration and size variation affected each other (Figure 12a). A uniform intensity increase of BS was observed in the entire sample area. Especially, an increase in BS was noticeably confirmed by the upper portion of the sample with time. After 5 min, the intensity increase in BS seen from the upper layer was also observed in the middle portion. This intensity gradation caused by titania particles in the middle and upper parts indicated a simultaneous concentration gradation and flocculation. Although it was challenging to distinguish particle agglomeration because of flocculation or coalescence by increase or decrease in the BS profile, it was determined by whether the initial particle size is 0.6 μm or less because of diffusion behaviors (Rayleigh or Mie). No overall concentration gradient existed from the lower to the upper area; therefore, synthesized titania particles did not completely float to the top surface. The migration phenomenon to the surface layer was only confirmed in the case of particles in the middle and upper sides, predominantly because of the decrease in the density of particles because of the voids (air) of the particles. The hollow structure induced the particles to migrate to the surface layer. No big differences existed between the particle sizes, shell thicknesses, and void fractions in this study. When micron-sized or sub-micron-sized particles were prepared, it was expected that the buoyant capability would be significantly reinforced.

Figure 12b,c shows top and cross-sectional images of the hollow titania pigmented coating. It determined the degree of floating to the surface because of the hollow structure by adding particles to the clear coating. The synthesized hollow titania particles were distributed as agglomerated forms within the coating film. Most particles were at the upper part of the coating film. However, they are non-uniform as a layer and are partially distributed in the coating. In Figure 12, the BS profile of Turbiscan assumed that synthesized particles were an aggregation shape in the solution. When the particle-pigmented coating was applied, they were observed as agglomerated particles in the coating film. The gathering phenomena were caused by the preparation method of particles produced through the sintering process. During heat treatment, it formed a network between titania particles because of the stabilizing properties of the nano-titania new crop. Here, the particle group had the disadvantages of floating onto the surface because of its high density. Hence, a technique ensuring dispersion stability between manufactured particles was needed. However, when sufficient buoyant performance was achieved, such as higher void fraction and dispersion stability between particles, that characteristically could support the movement to the surface layer as a driving force of the particles. A uniform layer could be formed on the surface of the coating film by attaining those requirements.

**Figure 13:**
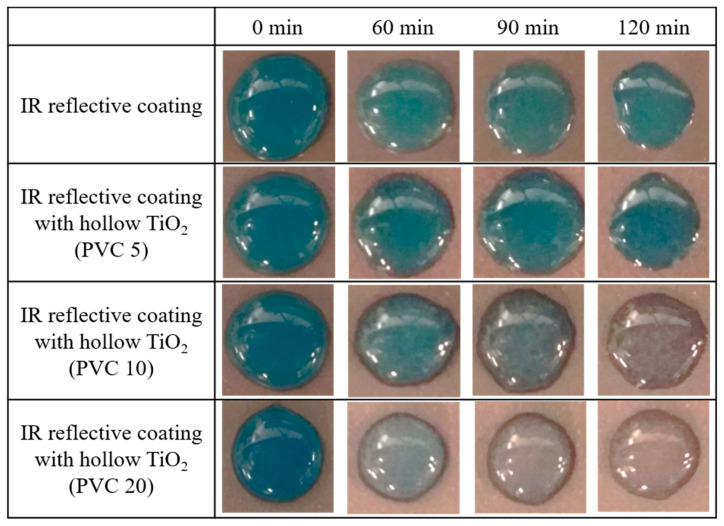
Visual images of methylene blue decomposition with UV exposure time.

It was intended to confirm the decomposition effect of organic substances by applying the prepared particles to commercial IR reflective coatings. Figure 13 shows the decomposition performance of organic dust (methylene blue) on a coated steel sheet under UV irradiation. Because titania nanoparticles are contained in the coating material, methylene blue can be decomposed by the photocatalytic effect. The content of titania particles was adjusted to the volume percentages of the paint and dispersed in the solvent of the paint. Samples with an input content of 5% of the total paint volume (PVC 5) were insufficient to show photoactivity. It is challenging to offer photocatalytic properties, even in the UV irradiation environment, if the photocatalyst is presented only in the coating or not sufficiently located near the coating surface, even though a good photocatalyst was applied on the coating. To overcome this, it is critical to increase the number of pigments or induce the particles near the surface layer. When synthesized hollow titania particles are applied, even with a small amount of PVC 10, it promotes the decomposition performance of methylene blue. After an exposure time of 2 h, most of the methylene blue was removed from the PVC 10 sample, revealing a clear aqueous solution. In the case of the PVC 20 sample, the photocatalytic effect of the particles showed excellent performance, and a large amount of methylene blue was removed after 60 min of UV irradiation. A clear aqueous solution was observed after 90 min of irradiation time. It is inferred that particles present near the surface layer can function as photocatalysts because of the floating characteristics of the hollow structure. Furthermore, the hollow structure increases the surface area compared to ordinary photocatalytic nanoparticles.

N-doped particles were produced because a base catalyst containing nitrogen, such as ammonia or DEMA, was used during synthesizing. The N-doped titania particles provide additional light absorption under the visible region, improving photocatalyst efficiency [10]. In this research, multiphase particles were produced that also accelerate photocatalytic performance during the heat treatment process. Because of three factors, better efficiency can be achieved by a small amount compared to the commercially available photocatalyst particles. To maximize this effect, photocatalyst particle distribution at the interface between the photoactive reaction environment and the coating surface is needed. Therefore, when a dense and uniform particle layer forms on the surface layer, this effect could be achieved even in a smaller amount.

**Figure 14:**
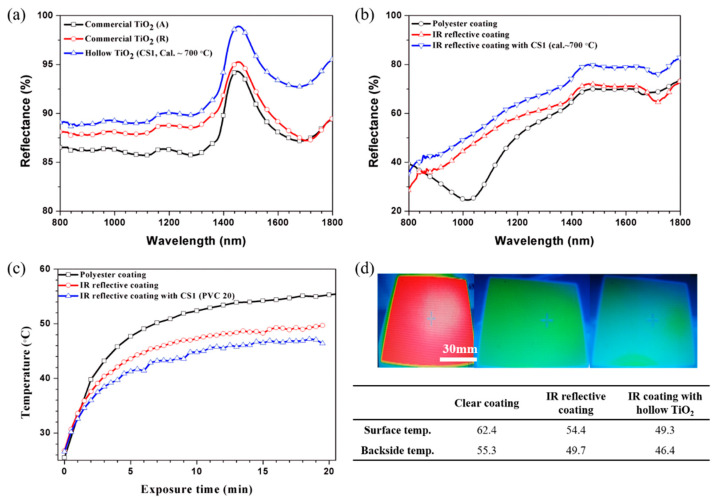
(**a**) UV–vis-NIR reflection spectrum of synthesized hollow particles; (**b**) their coating; (**c**) backside temperature profile of coated steel sheet with/without hollow titania, and (**d**) surface images measured by a thermal detection camera.

Figure 14 shows various heat shield abilities of particles and coatings. Reflectance measurement and near-IR wavelength were conducted to evaluate the reflection effect of the particles (Figure 14a,b). The reflectance spectrum of Figure 14a shows that the reflectance of the synthesized particles was higher in the near-infrared wavelength (*λ* > 800 nm) than in the anatase or rutile titania. Heat treatment was performed at 700 °C during synthesizing. The hollow titania was composed of multiphase (A:R ~84:16) titania. Therefore, for the effect of the phase of the particle, the reflectance should be lower than that of the rutile particle. However, because the reflectance of the hollow titania particle was measured higher, it is an result of the hollow structure. Zxing et al. [45] studied the effect of hollow silica particles on reflectance with different particle sizes, surface areas, and shell structures. An investigation on the effect of the hollow cavity size on solar reflectivity confirms that an increase in the HS size favors more reflection of longer *λ* light. Micron-sized well-desired hollow particles were required for maximizing scattering efficiency to incident light. A light reflection and refraction on the concave surface of the hollow chamber promote the overall reflectivity using 300 nm hollow silica. It was explained by light reflectivity versus the normal light particle interactions and involved only the external surface. Similar to hollow silica, the reflectivity of hollow titania (CS1, 200 nm) can be enhanced by increasing the reflection path because of cavity size. Furthermore, this effect is also exhibited in the case of the coating (Figure 14b). The reflectance of the coating in the NIR region has a similar increment tendency when particles are incorporated into the coating.

Finally, the thermal temperature profiling of a back-panel coated steel sheet under the heat source was measured. Heat transfer characteristics consist of a combination of four factors, namely, emissivity, convection, conduction, and reflection. In our research, the effect of convection was minimized by measuring the specimens simultaneously. Light reflection and heat transmission (conduction effect) were discussed in this study. The nature of IR reflective pigments in a coating induces light reflection and reduces the surface temperature of the coating. The surface temperature of the specimens was measured using a thermal imaging camera after 20 min in a heat source irradiation environment to compare the light reflection ability (Figure 14d). The surface temperature is determined by the reflection effect and emission of the coating. Compared to polyester clear coating, the heat shield paint and hollow titania-containing paint show excellent light-reflecting ability. As indicated above, adding titania increases the reflectance in the IR region. Hence, it shows an additional temperature reduction effect during actual measurement. In the case of commercial IR-reflective coated steel sheets, the backside temperature of the coated steel is 49.7 °C, whereas that of polyester clear coating is 55.3 °C after 20 min thermal irradiation. When the hollow titania particles were used, the heat shield ability was further strengthened to 46.4 °C. The temperature at the back of the specimen is the result of multiple factors of reflection, conduction, and emission, making it challenging to conclude that only one specific factor has influence. However, because the tendency of the surface temperature of the specimen appears similar on the back, the reflection effect prevails as the main factor in this coating system.

## 4. Conclusions

A study was conducted to prepare a novel hollow titania pigmented infrared reflective coating layer on steel substrate for better and prolonged performances. Surface contamination is a critical factor affecting the coating’s functionality and weatherability. Some functional paints, such as thermal barrier paints, dramatically decrease thermal control performance when organic pollutants, present in the atmosphere, are adsorbed on the coating surface.

This study used a hollow titania photocatalyst to achieve surface cleaning of the coating. It is more challenging to reveal the performance while the particles are in a binder rather than in a powder. When it presents near the coating layer, light can be easily absorbed, and contaminants adsorbed on the surface of the photocatalyst exhibit an adequate effect. Hollow structured titania was successfully produced and applied to the paint to achieve catalytic performance at 1-coat-1-bake. In this case, it is economically strengthened, and the unnecessary coating processes can be removed, so productivity is high. To manufacture the hollow particles, a direct chemical deposition method was used. Because the synthesis method proceeds in the heterophase, the titania precursor must be selectively formed on the target polymer template. A polymer template modified with MAA and titania with a core-shell structure were synthesized in the presence of a nitrogen-based base catalyst. At high pH, the polymer template has a negative charge on the surface by the carboxylate salt group, and the salt of the catalyst with a positive charge forms a salt on the polymer surface. The titania precursor is then selectively adsorbed to help the reaction.

To obtain a hollow structure, a process of removing the polymer core is required. In this study, heat treatment was conducted. The hollow titania particles formed as the heat treatment temperature was changed, confirming various A/R phase fractions. The different phase composition indicated another light absorption capability in a longer wavelength region, which is a critical characteristic of photocatalytic efficiency. In an organic decomposition test, CS1 particles (250 nm, calcinated at 700 °C) showed the best photocatalytic activity because of the proper phase composition between anatase and rutile. Furthermore, because of the hollow structure, it has a higher specific surface area than commercial photocatalyst particles, which means an increase in the active site, which is beneficial for improving photocatalyst efficiency.

The particle-pigmented coating also showed brilliant efficiency for both the removal of organic pollutants and the increase in IR reflectance. The particle floating tendency to the surface was examined, and the results show that hollow titania also promoted the performance of IR reflective coating.

## Figures and Tables

**Table 1:** Core-shell titania nanoparticle synthesis formulation.

	CS1	CS2	CS3
Core 1 solution	2.5 g		
Core 2 solution		2.5 g	
Core 3 solution			6.5 g
Ethanol	70 g	70 g	40 g
Titanium butoxide	4.0 g	4.0 g	4.8 g
DMEA	0.25 g	1.0 g	
Ammonia			0.2 g

**Table 2:** Formulation of polyester clear coating.

	Weight (g)
Polyester resin	20
Curing agent	1.45
Catalyst A	0.1
Catalyst B	0.5
Solvent	7
NV (%)	40

**Table 3:** The phase composition and crystallite size of hollow titania spheres.

	Surface Area (m^2^/g)	Pore Volume (cm^3^/g)	Crystallite Size (nm)	Phase Composition
Commercial TiO_2_	40.32	0.009		
CS1	71.44	0.156	20.73	96.6:3.34
CS2	52.24	0.116	19.76	91.1:8.9
CS3	89.496	0.169	18.88	99.0:1.0

**Table 4:** Phase composition of hollow titania via calcination temperature.

Calcination Temperature	CS1	CS2	CS3
400 °C	Amorphous	Amorphous	96.9:3.1
500 °C	99.04:0.96	98.94:1.06	98.81:1.19
600 °C	96.6:3.34	91.1:8.9	99.0:1.0
700 °C	84.21:15.79	73.31:26.69	98.83:1.17
800 °C	1.8:98.2	0.3:99.7	56.1:43.9

## Data Availability

The data presented in this study are available on request from the corresponding author.

## References

[1] Fujishima A., Honda K. (1972). Electrochemical Photolysis of Water at a Semiconductor Electrode. Nature.

[2] Maeda K. (2011). Photocatalytic Water Splitting Using Semiconductor Particles: History and Recent Developments. J. Photochem. Photobiol. C Photochem. Rev..

[3] Abe R. (2010). Recent Progress on Photocatalytic and Photoelectrochemical Water Splitting under Visible Light Irradiation. J. Photochem. Photobiol. C Photochem. Rev..

[4] Choi W., Termin A., Hoffmann M.R. (1994). The Role of Metal Ion Dopants in Quantum-Sized TiO_2_: Correlation between Photoreactivity and Charge Carrier Recombination Dynamics. J. Phys. Chem..

[5] Kim W., Seok T., Choi W. (2012). Nafion Layer-Enhanced Photosynthetic Conversion of CO_2_ into Hydrocarbons on TiO_2_ Nanoparticles. Energy Environ. Sci..

[6] Hoffmann M.R., Martin S.T., Choi W., Bahnemann D.W. (1995). Environmental Applications of Semiconductor Photocatalysis. Chem. Rev..

[7] Fujishima A., Zhang X., Donald T.A. (2007). Heterogeneous Photocatalysis: From Water Photolysis to Applications in Environmental Cleanup. Int. J. Hydrogen Energy.

[8] Yates H.M., Nolan M.G., Sheel D.W., Pemble M.E. (2006). The Role of Nitrogen Doping on the Development of Visible Light-Induced Photocatalytic Activity in Thin TiO_2_ Films Grown on Glass by Chemical Vapour Deposition. J. Photochem. Photobiol. Chem..

[9] Bessekhouad Y., Robert D., Weber J.-V., Chaoui N. (2004). Effect of Alkaline-Doped TiO2 on Photocatalytic Efficiency. J. Photochem. Photobiol. Chem..

[10] Asahi R., Morikawa T., Ohwaki T., Aoki K., Taga Y. (2001). Visible-Light Photocatalysis in Nitrogen-Doped Titanium Oxides. Science.

[11] Ohno T., Akiyoshi M., Umebayashi T., Asai K., Mitsui T., Matsumura M. (2004). Preparation of S-Doped TiO_2_ Photocatalysts and Their Photocatalytic Activities under Visible Light. Appl. Catal. Gen..

[12] Assadi M.H.N., Hanaor D.A.H. (2016). The effects of copper doping on photocatalytic activity at (101) planes of anatase TiO_2_: A theoretical study. Appl. Surf. Sci..

[13] Doustkhah E., Assadi M.H.N., Komaguchi K., Tsunoji N., Esmat M., Fukata N. (2021). In situ Blue titania via band shape engineering for exceptional solar H_2_ production in rutile TiO_2_. Appl. Catal. B Environ..

[14] Jana A.K. (2000). Solar Cells Based on Dyes. J. Photochem. Photobiol. Chem..

[15] Dhanalakshmi K.B., Latha S., Anandan S., Maruthamuthu P. (2001). Dye Sensitized Hydrogen Evolution from Water. Int. J. Hydrogen Energy.

[16] Rabatic B.M., Dimitrijevic N.M., Cook R.E., Saponjic Z.V., Rajh T. (2006). Spatially Confined Corner Defects Induce Chemical Functionality of TiO2 Nanorods. Adv. Mater..

[17] Saponjic Z.V., Dimitrijevic N.M., Tiede D.M., Goshe A.J., Zuo X., Chen L.X., Barnard A.S., Zapol P., Curtiss L., Rajh T. (2005). Shaping Nanometer-Scale Architecture Through Surface Chemistry. Adv. Mater..

[18] Antonelli D.M., Ying J.Y. (1995). Synthesis of Hexagonally Packed Mesoporous TiO_2_ by a Modified Sol–Gel Method. Angw. Chem. Int. Ed. Engl..

[19] Zheng Z., Huang B., Qin X., Zhang X., Dai Y. (2010). Strategic Synthesis of Hierarchical TiO_2_ Microspheres with Enhanced Photocatalytic Activity. Chem. Eur. J..

[20] Wang Y., Zhang L., Deng K., Chen X., Zou Z. (2007). Low Temperature Synthesis and Photocatalytic Activity of Rutile TiO_2_ Nanorod Superstructures. J. Phys. Chem. C.

[21] Wu J.-M., Zhang T.-W., Zeng Y.-W., Hayakawa S., Tsuru K., Osaka A. (2005). Large-Scale Preparation of Ordered Titania Nanorods with Enhanced Photocatalytic Activity. Langmuir.

[22] Yu J., Dai G., Cheng B. (2010). Effect of Crystallization Methods on Morphology and Photocatalytic Activity of Anodized TiO_2_ Nanotube Array Films. J. Phys. Chem. C.

[23] Kondo Y., Yoshikawa H., Awaga K., Murayama M., Mori T., Sunada K., Bandow S., Iijima S. (2008). Preparation, Photocatalytic Activities, and Dye-Sensitized Solar-Cell Performance of Submicron-Scale TiO_2_ Hollow Spheres. Langmuir.

[24] Li H., Bian Z., Zhu J., Zhang D., Li G., Huo Y., Li H., Lu Y. (2007). Mesoporous Titania Spheres with Tunable Chamber Structure and Enhanced Photocatalytic Activity. J. Am. Chem. Soc..

[25] Song C., Wang D., Gu G., Lin Y., Yang J., Chen L., Fu X., Hu Z. (2004). Preparation and Characterization of Silver/TiO_2_ Composite Hollow Spheres. J. Colloid Interface Sci..

[26] Jokanović V., Spasić A.M., Uskoković D. (2004). Designing of Nanostructured Hollow TiO2 Spheres Obtained by Ultrasonic Spray Pyrolysis. J. Colloid Interface Sci..

[27] Farrusseng D., Aguado S., Pinel C. (2009). Metal–Organic Frameworks: Opportunities for Catalysis. Angew. Chem. Int. Ed..

[28] Li R., Hu J., Deng M., Wang H., Wang X., Hu Y., Jiang H.-L., Jiang J., Zhang Q., Xie Y. (2014). Metal–Organic Frameworks: Integration of an Inorganic Semiconductor with a Metal–Organic Framework: A Platform for Enhanced Gaseous Photocatalytic Reactions. Adv. Mater..

[29] Caruso F., Caruso R.A., Möhwald H. (1998). Nanoengineering of Inorganic and Hybrid Hollow Spheres by Colloidal Templating. Science.

[30] Yu J., Xiang Q., Ran J., Mann S. (2009). One-step hydrothermal fabrication and photocatalytic activity of surface-fluorinated TiO_2_ hollow microspheres and tabular anatase single micro-crystals with high-energy facets. Crystengcomm.

[31] Akbari H., Pomerantz M., Taha H. (2001). Cool Surfaces and Shade Trees to Reduce Energy Use and Improve Air Quality in Urban Areas. Sol. Energy.

[32] Ianoş R., Muntean E., Păcurariu C., Lazău R., Bandas C., Delinescu G. (2017). Combustion Synthesis of a Blue Co-Doped Zinc Aluminate near-Infrared Reflective Pigment. Dye. Pigment..

[33] Meenakshi P., Selvaraj M. (2018). Bismuth Titanate as an Infrared Reflective Pigment for Cool Roof Coating. Sol. Energy Mater. Sol. Cells.

[34] Soumya S., Mohamed A.P., Paul L., Mohan K., Ananthakumar S. (2014). Near IR Reflectance Characteristics of PMMA/ZnO Nanocomposites for Solar Thermal Control Interface Films. Sol. Energy Mater. Sol. Cells.

[35] Yang Z., Xue X., Dai J., Li Y., Qin J., Feng Y., Qu J., He Z., Sun P., Xu L. (2018). Study of a super-non-wetting self-cleaning solar reflective blue-grey paint coating with luminescence. Sol. Energy Mater. Sol. Cells.

[36] Wen G., Guo Z., Liu W. (2017). Biomimetic Polymeric Superhydrophobic Surfaces and Nanostructures: From Fabrication to Applications. Nanoscale.

[37] Zhang J., Zhu C., Lv J., Zhang W., Feng J. (2018). Preparation of Colorful, Infrared-Reflective, and Superhydrophobic Polymer Films with Obvious Resistance to Dust Deposition. ACS Appl. Mater. Interfaces.

[38] Rauf M.A., Meetani M.A., Hisaindee S. (2011). An overview on the photocatalytic degradation of azo dyes in the presence of TiO_2_ doped with selective transition metals. Desalination.

[39] Hong J., Han H., Hong C.K., Shim S.E. (2008). A Direct Preparation of Silica Shell on Polystyrene Microspheres Prepared by Dispersion Polymerization with Polyvinylpyrrolidone. J. Polym. Sci. Part A Polym. Chem..

[40] Chen M., Zhou S., Wu L., Xie S., Chen Y. (2005). Preparation of Silica-Coated Polystyrene Hybrid Spherical Colloids. Macromol. Chem. Phys..

[41] Schmid A., Fujii S., Armes S.P. (2006). Polystyrene−Silica Nanocomposite Particles via Alcoholic Dispersion Polymerization Using a Cationic Azo Initiator. Langmuir.

[42] Wang P., Chen D., Tang F.-Q. (2006). Preparation of Titania-Coated Polystyrene Particles in Mixed Solvents by Ammonia Catalysis. Langmuir.

[43] Niu Z., Yang Z., Hu Z., Lu Y., Han C.C. (2003). Polyaniline–Silica Composite Conductive Capsules and Hollow Spheres. Adv. Funct. Mater..

[44] Shi L., Wang X., Lu L., Yang X., Wu X. (2009). Preparation of TiO_2_/polyaniline nanocomposite from a lyotropic liquid crystalline solution. Synth. Met..

[45] Xing Z., Tay S.-W., Ng Y.H., Hong L. (2017). Porous SiO_2_ Hollow Spheres as a Solar Reflective Pigment for Coatings. ACS Appl. Mater. Interfaces.

